# The relationship between lifecourse traumatic events and pain in an older rural South African population: A cross-sectional study

**DOI:** 10.1371/journal.pone.0313140

**Published:** 2024-12-16

**Authors:** Ting Ting Wang, Collin Payne, Sumaya Mall, Stephen Tollman, Guy Harling

**Affiliations:** 1 School of Medicine, National Yang Ming Chiao Tung University, Taipei, Taiwan; 2 Department of Medical Education, Taipei Veterans General Hospital, Taipei, Taiwan; 3 Institute for Global Health, University College London, London, United Kingdom; 4 School of Demography, Research School of Social Sciences, The Australian National University, Canberra, ACT, Australia; 5 Harvard Center for Population and Development Studies, Harvard University, Cambridge, Massachusetts, United States of America; 6 Department of Epidemiology and Biostatistics, University of the Witwatersrand, Johannesburg, South Africa; 7 MRC/Wits Agincourt Unit, Rural Public Health and Health Transitions Research Unit (Agincourt), University of the Witwatersrand, Johannesburg, South Africa; 8 International Network for the Demographic Evaluation of Populations and their Health Network, Accra, Ghana; 9 Africa Health Research Institute, KwaZulu-Natal, South Africa; 10 School of Nursing & Public Health, College of Health Sciences, University of KwaZulu-Natal, Durban, South Africa; Ahmadu Bello University, NIGERIA

## Abstract

**Background:**

Pain in older adults is an increasing concern in low- and middle-income countries (LMICs), with literature suggesting an association with past traumatic events (TEs) in high-income settings. We aim to investigate this relationship in a population-representative sample of older adults with high burden of TEs in a rural South African community.

**Methods:**

The Health and Aging in Africa: A longitudinal Study of an INDEPTH Community in South Africa (HAALSI) study collected data pain intensity, using the Brief Pain Inventory, and TEs with a 16-item questionnaire, from 2411 participants aged 40–79 in 2014–15. We used logistic regression models to test the association between TE exposure and self-reported pain status.

**Results:**

TE experience was near-universal (99.1% experience of at least one), while 9.0% of participants reported current pain, of which 86.6% was moderate/severe. In multivariable regression, increased odds of moderate/severe pain was associated with more TEs of any kind (OR 1.08; 95%CI 1.02–1.15 per additional TE) and with past exposure to disasters, accidents and illnesses (men and women), violence in the community (women only) and social/family environment problems (men only)–but not with childhood or war-related TEs.

**Conclusions:**

TEs were associated with pain even within a rural resource-limited setting where trauma experiences were extremely common. However, associations varied by TE type and sex. Interventions to prevent pain in older adults need to be targeted to block specific mechanisms that vary within even at-risk populations.

## Introduction

Pain, as defined by International Association for the Study of Pain (IASP) is an adverse sensory and emotional experiences potentially associated with tissue damage [1]. However, the notion has shifted from a protective response to a pathologic disease state in recent years [2]. Pain was classified as acute or chronic based on duration [3]. At least 10% of the world’s population is estimated to suffer from chronic pain [4], a figure projected to increase as the world’s population ages [5]. Chronic pain co-occurs with non-communicable diseases (NCDs), such as mental disorders, diabetes mellitus, arthritis and cancer, whose burden also is increasing, leading to more comorbidities [6,7]. Older populations with high rates of mulitmorbidity, are disproportionally more prone to suffer chronic pain [8]. The consequences of chronic pain are substantial: lower back pain alone is the greatest contributor to worldwide years lived with disability (YLDs) [9]. Although chronic pain is a worldwide concern, evidence on chronic pain and its risk factors in low and middle-income countries (LMICs) is more limited than in high-income countries; much LMIC pain research has focused around HIV, post-operative pain and sickle cell disease [10–13].

Acute pain, with a clear causality such as musculoskeletal injuries, is typically short-term [14]; Chronic pain, however, is believed to be predicted by the combination of biological, psychological and social factors [8,15–17]. While biological and psychological risks factors for chronic pain development are relatively well-researched, social perspectives on pain are gradually receiving more attention–not least because social determinants of NCDs, such as low socioeconomic status, educational and poverty can also be associated with pain [6,16]. One important aspect of social risk factors is that of traumatic events (TEs). While most studies focused on TEs in childhood (i.e., adverse childhood experiences) as a critical period for neurodevelopment and human capital development [18], there is increasing evidence that TEs experienced throughout the lifecourse may be associated with negative health outcomes [19]. TEs, especially if chronic or severe, can generate biopsychological effects, including physcial and mental health conditions [20–22].

Adults exposed to TEs have higher odds of future chronic pain, including headache or migraine, back and neck pain, chronic pelvic pain, chronic widespread pain and fibromyalgia [20,23–26]. Much existing literature on this relationship is cross-sectional and retrospective. However, a UK longitudinal analysis demonstrated that TEs in childhood including family financial hardship, experiencing institutional care and maternal death were each associated with increased chronic widespread pain even after adjustment for social class and psychological distress [27].

Several mechanisms linking TEs and later-life pain have been proposed, including biological (e.g., dysregulation of allostatis) and psychological (e.g., psychopathology) ones [17]. Allostatic overload, a harmful response to TEs, can manifest as chronic inflammation throughout the life course [28,29], increasing the risk of chronic pain both directly and by promoting development of chronic NCDs [30]. Psychopathology, notably anxiety, depression and post-traumatic stress disorder (PTSD), can also mediate the relationship between TEs and chronic pain [30–33].

Despite much work in higher-income settings, little is known about the impact of TEs on pain in LMICs. Associations might be expected to differ in LMICs for several reasons. First, there is evidence that TEs are prevalent in LMICs, including Mexico, the Philippines, Malawi and South Africa [34–37]. Second, the relative frequencies of TEs likely differ, e.g., war and conflict-related experiences may be more prevelant in LMICs [38]. Third, NCDs develop on average at earlier ages among populations in LMICs than in HICs [39,40]. South Africa typifies all these differences. The majority Black African population experienced apartheid from 1948 to 1994, with resulting exposure to political and economic deprivation which led to near-universal traumatic experiences, often violence-related (both witnesses and experiences)– 88% of the population-representative Birth to 20+ cohort in the Gauteng Province of South Africa reported at least one TEs by early adulthood [41,42]. These experiences have been linked to increased likelihood of physical and psychological ill-health in older age [19]. Some settings in northern and eastern South Africa also accommodate large number of refugees and migrants from violence beyond the country’s borders, e.g., from the Mozambican Civil War in the 1980s and Zimbabwe’s economic troubles of the past two decades and more.

Despite this high burden of TEs, and a national adult prevalence of chronic pain estimated at 18.3% [43], the association between TEs and pain is little-studied. One past study using the South Africa Stress and Health study (2002–05) with a nationally representative sample of over-15-year olds found a dose-response association between cumulative count of TEs and chronic pain [42]. However, it is likely both that associations between TEs and pain will differ both at older ages, especially as overall health conditions increase with age, and by rurality, especially given likely higher levels of TE and pain in former Apartheid homelands. In this work, we seek to understand the connection between TEs and pain in a context where TE exposure is near universal. We focus on current pain as a reflection of both acute and chronic pain, acknowledging it may not only capture the chronicity in a large cohort of older adults (aged 40+ in 2015) living in rural Mpumalanga province, South Africa. One-third of this cohort moved to the area as refugees from Mozambique due to its 1977–1992 civil war–an experience linked with substantive long-term trauma for many who lived through it [44]–while the remainder grew up under Apartheid. Both experiences generated a wide range of traumatic experiences that have the potential to have life-long repercussions, including pain. While this context of two potentially overlapping sources of societal trauma may be near-unique, we expect our analyses to identify how patterns of TEs affect later-life pain in settings where almost everyone has experienced some TEs.

## Materials and methods

### Study setting and sample

Health and Aging in Africa: a Longitudinal Study of an INDEPTH community in South Africa (HAALSI), was initiated within the established Agincourt health and socio-demographic surveillance system (HDSS) located in Mpumalanga province, notheastern South Africa [45]. The Agincourt HDSS is located within the former homeland of Gazankulu, with a long history of underprovision of public services, very high unemployment and high prevalence of HIV and other health conditions. Due to its proximity to Mozambique, around one-third of the local population are Mozambican migrants and their descendants, many of who arrived during the Mozambican Civil War from 1977 to 1992 [46].

HAALSI randomly sampled 6281 HDSS-resident adults aged over 40 on 1 July 2014, of whom 5059 were found and consented to participate in 2014–15 [45]. Face-to-face interviews were conduncted by experienced local field workers using table computers; interviews comprised household, individual questionnaries, anthropometric measurements and blood draws. Our analytic sample utilised cross-sectional data from the baseline wave of HAALSI and comprised the random subsample of HAALSI respondents aged 40 to 79 who were invited to complete additional activities, including an in-depth life-history questionnaire and laboratory visit; participants did not differ substantially from same-aged non-participants [19].

### Ethical approval

Ethical approval for HAALSI was obtained from the University of the Witwatersrand Human Research Ethics Committee, the Harvard T.H. Chan School of Public Health Office of Human Research Administration, and the Mpumalanga Provincial Research and Ethics Committee. HAALSI participants provided written informed consent prior to participation. Participants who could not read had a signed witness assisted them and provided an inked fingerprint as signature. The data used in this analysis was anonymised and publicly available, and thus did not require additional ethical approval. The datasets generated and analysed during the current study are available in the HAALSI Baseline Survey repository, https://doi.org/10.7910/DVN/F5YHML, which we accessed on 16^th^, January, 2020.

### Measures

#### Outcomes

Pain intensity was assessed by the short-form version of Brief Pain Inventory, a 15-item self-reported questionnaire, measuring: 1) pain intensity; 2) pain location; and 3) the interference in life and well-being [47]. If respondents reported any pain today, they were asked four pain intensity questions, each on an 11-point scale ranging from 0 (no pain) to 10 (pain as bad as you can imagine). These questions covered the worst, least and average pain over the past 24 hours, and pain level right now, but not the duration of pain. A similar pain questionnaire, the Wisconsin Brief Pain Questionnaire [48] with the same questions regarding pain intensity had been validated in Xitsonga/Shangaan, the local language [49]. The HAALSI questions were also back-translated for reliability.

Our primary outcome measure was the intensity of ‘average pain’, as it implicity reflects the persistent of pain, with ‘pain now’ included as a sensitivity analysis. We categorised ‘average pain’ intensity into four levels: none (0); mild (1–3), moderate (4–6) and severe (7–10) in line with widely used cutpoints [50,51]. In regression analysis, we further divided responses into no/mild and moderate/severe pain, based on mild pain’s relatively low impact on daily function and health-related quality of life [52,53].

#### Exposures

The TE questions in HAALSI were derived from the life-history section in the English Longitudinal Study of Aging (ELSA) [54]. The questionnaire comprised yes-no questions regarding experience of 16 adversities (wording given in Table 2). We coded individuals skipping individual questions as ‘don’t know/refuse to answer’ unless they had skipped eight or more TEs, in which case they were dropped from our analysis. We grouped TEs into five categories based on previous trauma studies: childhood household dysfunction; social/family environment; violence in the community; natural disasters, accidents and illnesses; and war-related events [19,55]–since each type of traumatic experience may have different effects on mental and physical health [56]. We separated war-related events from violence in the community to underscore the influences of the Mozambican Civil War.

We used two exposure variables. First, we calculated the cumulative number of TEs by summing the number of affirmed events (from 0 to 16), treating “don’t know” answers as non-affirmative. This parameterisation was based on the cumulative risk hypothesis which expects risk factors to accumulate and to increase the possibility of later-life negative health outcomes [29,57]. Second, to examine the impacts of specific TE types, we created five binary variables to indicate any TE experience in each TE category. A TE category was deemed affirmative if any one item was affirmed; any respondent who did not affirm any response in a TE category, but answered “don’t know” to at least one question, were grouped separately as “any don’t know”. This parameterisation assumed that any TE in a category was equally important and that a single exposure would have as much effect as multiple types within a category. Overall, the parameterisation mirrored earlier analysis of the same data for a broad range of mental, physical and cognitive health outcomes [19]. We chose not to regress individual traumatic events to avoid the risk of multiple testing.

#### Covariates

We identified several potential confounders using a directed acyclic graph focused on our exposure-outcome relationship (see S1 Fig in S1 File). We controlled for age and country of origin (South Africa and Mozambique/other) since TE exposure and pain experiences may differ across birth cohort and by past experiences such as civil war and Apartheid [19,55,58]. We also controlled for sex given past evidence of differences in reporting and perceiving pain, as well as in TE exposure risk [55,59]. Finally, we controlled for childhood socioeconomic status via the proxy measure of father’s occupation [60,61], categorised into two levels (i.e., skilled and unskilled) according to International Standard Classification of Occupations 2008 (ISCO-08) definitions [62,63].

### Data analysis

We excluded any respondent missing 50% or more of TE responses (n = 25), respondents aged under 40 (n = 8) or over 80 (n = 27), those missing country of origin (n = 1) and those not answering the ‘pain today’ question (n = 25). All remaining respondents were included in a complete case analysis.

First, we summarised the distribution of baseline characteristics, count of TEs and pain intensity using frequencies and proportions. We then conducted bivariate and multivariable logistic regression analyses for both exposure parameterisations. For all regressions, we grouped”any don’t know” TE values with those without exposure due to small numbers. First, we assessed dose-response relationships between TEs and the binary pain outcome. Second, we assessed the association of each TE category with pain. Third, we stratified our sample into men and women and reran our regression analyses for sex-differences in perception of pain, processing pain [59] and exposure to TEs [55], in order test for potential effect-modification by sex. We conducted three sensitivity analyses. First, we used ordinal logistic regression to assess whether results differed when looking at pain as a four-level outcome. Second, we added a quadratic TE count term to assess whether this improved model fit using the Akaike Information Criterion (AIC) and predicted probability plots. Third, we stratified our sample by country of origin (South Africa and Mozambique/other) and conducted regression analyses to assess whether pain outcomes varied between these distinct settings. All analysis was conducted in R version 3.6.1 [64].

## Results

**Fig 1:**
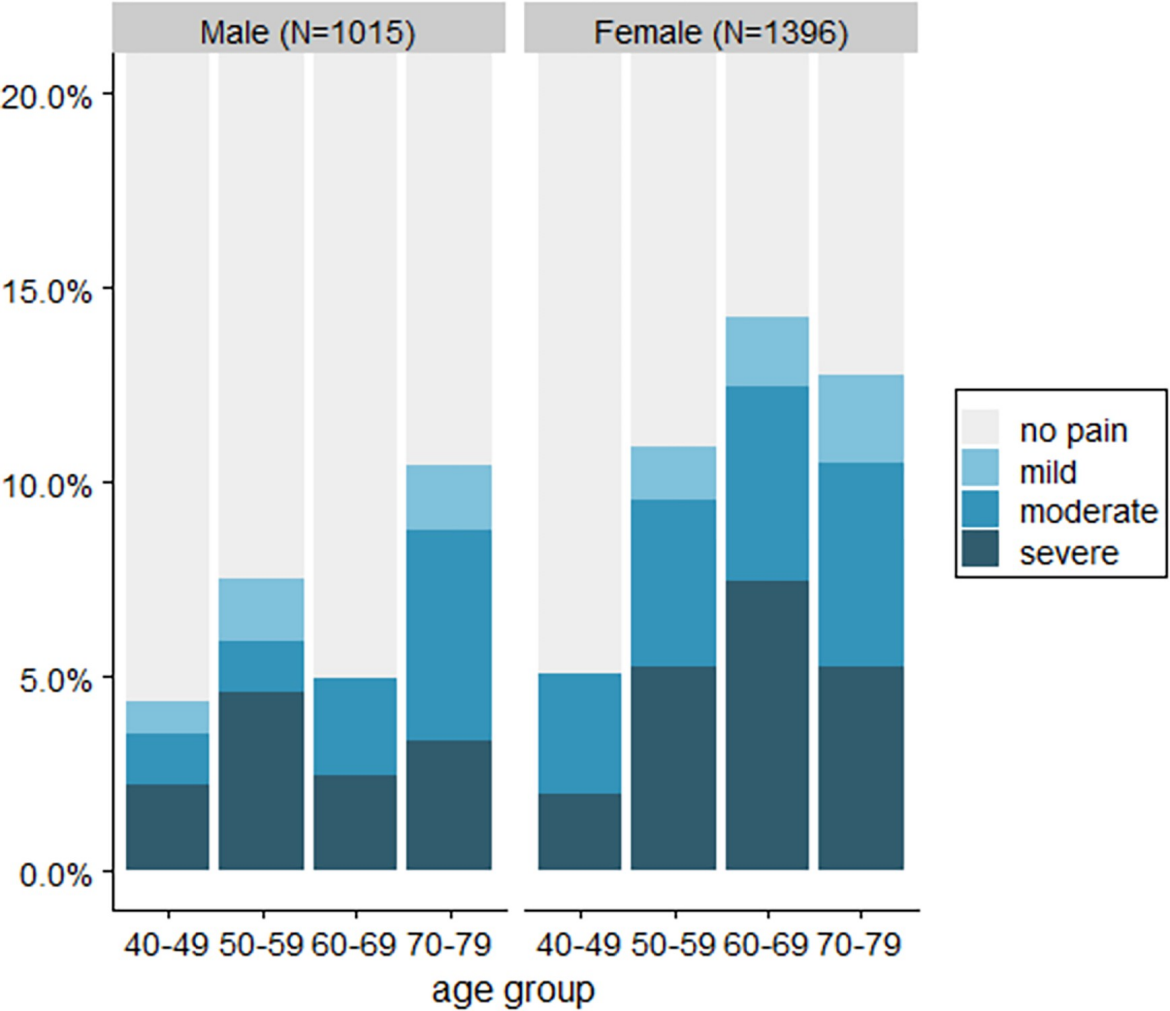
Proportion of respondents reporting pain intensity by sex and age.

We included 2411 of 2492 respondents who consented to the life history module (96.7%) as a complete case analysis (Table 1). Respondents were more likely to be female but were relatively balanced by age. Almost 30% were born outside of South Africa, almost all in Mozambique, and over half of respondents’ fathers had had a skilled occupation. Overall pain prevalence was 9.0%, with the great majority (86.6%) of these reporting moderate or severe pain. Pain generally rose with age and was most frequent for women aged 60–69 (Fig 1).

**Table 1:** Descriptive characteristics of HAALSI life history cohort (N = 2411).

	N (%)
**Sex**	
** Male**	1015 (42.1)
** Female**	1396 (57.9)
**Age**	
** 40–49**	582 (24.1)
** 50–59**	800 (33.2)
** 60–69**	522 (21.7)
** 70–79**	507 (21.0)
**Father’s occupation**	
** Skilled**	1217 (50.5)
** Unskilled**	694 (28.8)
** Other**	263 (10.9)
** Unknown**	237 (9.8)
**Country of origin**	
** South Africa**	1721 (71.4)
** Mozambique/other**	690 (28.6)
**Count of traumatic events**	
** 0 TEs**	21 (0.9)
** 1 TE**	150 (6.2)
** 2 TEs**	202 (8.4)
** 3 TEs**	311 (12.9)
** 4 TEs**	384 (15.9)
** 5+ TEs**	372 (15.4)
**Pain intensity**	
** No pain**	2195 (91.0)
** Mild**	29 (1.2)
** Moderate**	86 (3.6)
** Severe**	101 (4.2)

**Fig 2:**
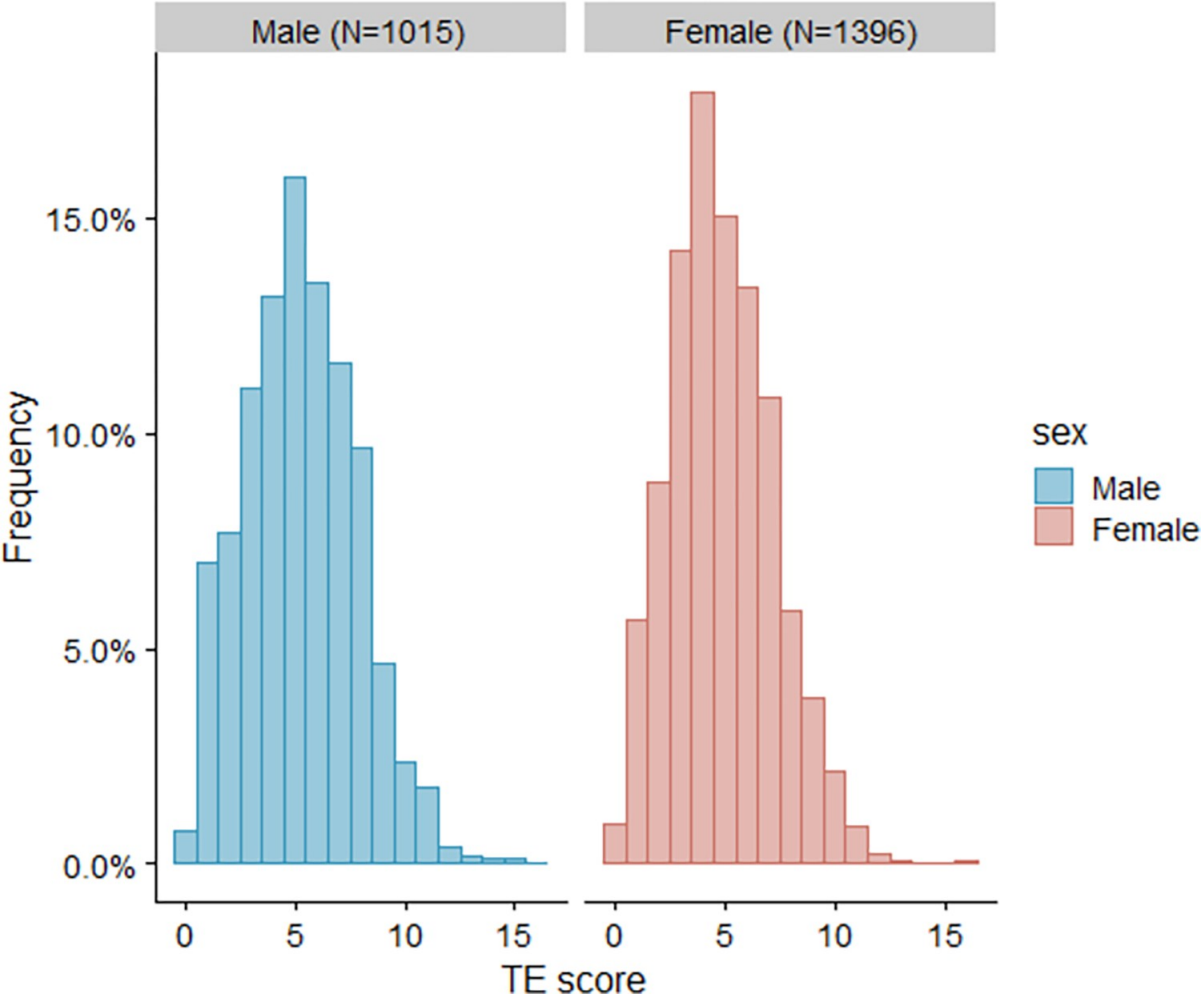
The distribution of traumatic events count, stratified by sex.

Almost all respondents (99.1%) had experienced at least one TE, with modal class of four for women and five for men, and wide variation (Fig 2). The most common TE was severe financial hardship experience (82.6%), followed by risk of death or death from accidents for close friends or relatives (67.4%); the least common TE was sexual assault (2.1%). The commonest TE categories were negative social and family environment (87.9%) and exposure to natural disaster, accident, and illness (87.3%); the least common was war-related events (23.1%). The most common unknown TEs were those for parental experiences within childhood household dysfunction (0.5–7.7% don’t know), while items relating directly to respondents were almost always known (≤0.7% don’t know) (Table 2).

**Table 2:** Reported lifecourse traumatic events by item and category (N(%)).

	Items		Categories	
	Affirmative	Don’t know	Any affirmative	Any don’t know^†^
**Childhood household dysfunction**			1419 (58.9)	62 (2.6)
Parents unemployed more than 6 months	328 (13.6)	186 (7.7)		
Parents often argued and fought	559 (23.2)	149 (6.2)		
Parental substance use and mental disorder	585 (24.3)	107 (4.4)		
Physical abuse from parents	905 (37.5)	11 (0.5)		
**Social*/*family environment**			2119 (87.9)	2 (0.1)
Husband, wife, partner, or child addicted to drugs or alcohol	510 (21.2)	1 (0.0)		
Provided long term-care to a disabled relative/friend	512 (21.2)	0 (0.0)		
Experienced severe financial hardship	1991 (82.6)	2 (0.1)		
**Violence in the community**			1491 (61.8)	3 (0.1)
Sexual assault (include rape or harassment)	51 (2.1)	4 (0.2)		
Physical attack or assault	636 (26.4)	4 (0.2)		
Witnessed serious accidents or violent acts	1190 (49.4)	0 (0.0)		
**Natural disaster, accident and illness**			2105 (87.3)	1 (0.0)
Major fire, flood, earthquake and other natural disaster	1160 (48.1)	2 (0.1)		
Life-threatening illness or accident	1223 (50.7)	3 (0.1)		
Close friends/relatives died/at risk of death from illness/accident	1625 (67.4)	2 (0.1)		
**War-related**			558 (23.1)	13 (0.5)
Fired a weapon	119 (4.9)	0 (0.0)		
Witnessed serious injury or death in war	370 (15.3)	2 (0.1)		
Lost close friends or relatives in war	333 (13.8)	17 (0.7)		

Note: †”Any don’t know “respondents are those with no affirmative response, but at least one “don’t know” response, in a category.

In bivariate analysis, each additional TE was associated with an 8% increase (95% confidence interval (CI) 1.02–1.15) in the odds of reporting moderate/severe pain (Table 3); this was little changed after adjustment for potential confounders or stratification by sex. In regressions for TE categories, two categories–violence in the community and disasters, illnesses and accidents–were associated with significantly increased odds of moderate/severe pain, with odds ratios of 1.62 (95%CI 1.17–2.27) and 1.70 (95%CI 1.01–3.05) respectively among all respondents. In sex-stratified models, pain was slightly more strongly associated with disasters, illnesses and accidents in women than men (OR 1.81; 95%CI 0.93–3.98 vs OR 1.56; 95%CI 0.70–4.00) but much more strongly associated with violence in the community for women than men (OR 1.93; 95%CI 1.30–2.90 vs OR 1.14; 95%CI 0.64–2.12). In contrast, adverse social/family environment was markedly more strongly associated with pain for men than women (OR 1.80; 95%CI 0.77–5.31 vs OR 0.97; 95%CI 0.56–1.78).

**Table 3:** Logistic regressions of moderate and severe pain with exposure to TE score and TE categories.

	Bivariate analysis	Multivariable analysis		
	OR (95% CI)	AOR (95% CI)		
		Total	Female	Male
**Traumatic event count**	1.08 (1.02–1.15)	1.09 (1.03–1.16)	1.09 (1.01–1.18)	1.09 (0.98–1.21)
**Childhood household dysfunction**	0.91 (0.67–1.23)	1.00 (0.73–1.36)	0.91 (0.63–1.32)	1.26 (0.72–2.28)
**Social/family environment**	1.16 (0.73–1.94)	1.18 (0.74–1.97)	0.97 (0.56–1.78)	1.80 (0.77–5.31)
**Violence in the community**	1.49 (1.08–2.07)	1.62 (1.17–2.27)	1.93 (1.30–2.90)	1.14 (0.64–2.12)
**Natural disaster, illness and accident**	1.60 (0.98–2.82)	1.70 (1.01–3.05)	1.81 (0.93–3.98)	1.56 (0.70–4.00)
**War-related**	0.99 (0.69–1.40)	1.06 (0.71–1.57)	1.09 (0.64–1.80)	1.06 (0.56–1.93)

Note: Results are from twenty-four separate logistic regressions. Reference group in all cases is the combination of ‘did not experience any TE in the category’ and those with ‘any don’t know’ answers in that TE category. Multivariable models were adjusted for age, sex, country of origin and father’s occupation, except when stratified on sex (full models shown in S3-S8 Tables in S1 File). OR = odds ratio; AOR: Adjusted odds ratio; CI = confidence interval.

The sensitivity analysis using four levels of pain did not give qualitatively different results from the two-level models (see S1 Table in S1 File). The quadratic TE model fit the data slightly better (AIC 1286.3 vs 1289.1 for the linear model), suggesting a possible decline in pain above eight TEs; however given the small number of individuals with such high values this inflection was highly uncertain (see S2 Fig in S1 File). The stratified analyses by country of origin showed small differences in the association between pain and TE categories, except for a weaker association with war-related events in respondents from Mozambique/other (OR 1.52 95%CI 0.91–2.47 vs OR 0.71 95%CI 0.39–1.25) (see S2 Table in S1 File).

## Discussion

Among older adults (ages 40 to 79) in rural Mpumalanga, South Africa, 9% of respondents reported current pain, with higher rates at older ages and for women. Moderate/severe pain prevalence rose with increasing numbers of reported traumatic events. Exposure to illnesses, accidents and disasters (men and women), violence in the community (women) and social/family environment problems (men) were most strongly associated with later pain. The study population has no doubt experienced political oppression and economic deprivation as children under Apartheid and one-third were civil war refugees from Mozambique, and thus a unique cohort to explore the connection between TEs and pain.

Pain prevalence in HAALSI was lower than in existing nationally representative South African studies: chronic pain was reported by 24.1% of Mpumalanga respondents in the 2016 South African Demographic and Health Survey (SADHS) [43], and by 46.6% of South Africa Stress and Health (SASH) study respondents [42]. The wide variation in pain prevalence may in part be a function of inconsistent definitions of pain and its chronicity. Both SADHS and SASH asked about a range of experiences of pain and discomfort, which might capture perceptions other than pain. HAALSI’s use of the Brief Pain Inventory, comprising questions about pain sites, intensity and life interference, may have helped provide a systematic measurement of pain via visualization, rather than capturing discomfort. However, the pattern of pain prevalence seen in HAALSI was similar to that elsewhere, with women and older individuals reporting more pain [8,59].

Our findings of a positive association between cumulative TE exposure and later-life pain are consistent with prior research in both high-income settings and LMICs [42,65,66]. This association is congruent with accumulation of risk models from lifecourse theory that hypothesise the negative effects of repeated exposures [67,68]. Potential biological and psychological pathways in support of these theories have been demonstrated: a dose-response relationship (i.e., accumulation) between cumulative trauma in childhood and inflammatory biomarkers (e.g., C-reactive protein) in middle-aged UK cohorts [29,69], and more widely with mental health issues, especially PTSD among older populations [19,70]. Our quadratic sensitivity analysis suggested that more TEs beyond some substantial number may add no additional risk of pain, or even reduce it. However, the substantial uncertainty around the estimate at high number of TEs warrants caution and further exploration.

Some TEs were more strongly associated with pain than others in HAALSI. Our findings align with existing evidence that exposure to violence (first-hand or witnessed) is associated with mental health conditions, somatic complaints or chronic pain in high-income settings [71–74] and LMICs [75]. Despite an abundance of literature on violence and pain, most studies have focused on psychological impacts–especially from intimate partner violence; our findings highlight that a wider range of violence types can predict physical pain. We also found stronger associations between violence and pain in females. This may reflect South African females’ greater experience of domestic and sexual violence, and less criminal assault, than South African men [76,77]–when linked to evidence that sexual abuse may be most pathogenic violence to mental health and chronic pain among females [74].

The significant association seen between illnesses, accidents and disaster and later-life pain tallies with previous evidence that experiencing disasters increases risks of subsequent physical and mental health, including pain in many settings [27,78–80]. Some chronic pain may reflect physical sequelae of life-threatening injury or accident, such as fractures in earthquake survivors [78]. But traumas appraised as serious may also generate chronic pain via various mental pathways of threat experience. These include uncertainty about safety of self or loved ones [81], and negative life events such as job loss [82], social network disruption [83], displacement [84] and bereavement. Both uncertainty and negative event pathways can lead to autonomic arousal and hypervigilance, and thus PTSD and chronic pain [85]. The sudden change and aspects of loss relating to negative events are stressors for all, but the elderly can find adaption particularly hard [86].

We found social and family environment to be non-significantly associated with higher odds of later-life pain in males but not females. While the mechanism for this difference is not entirely clear, it is notable that long-term care provision (one of the three items in this category) has been found to be associated with depression, stress, chronic illness, health decline and pain [87,88]. These associations are particularly strong in households with food insecurity and low income [89], which describes much of the Agincourt population. In South Africa, male migration for work leads to a particular emphasis on women providing care for children and the ill, especially where HIV is highly prevalent [90], generating greater caregiver burden for women [91]. The stronger association between family TEs and pain in males may reflect South Africa women considering long-term caregiving as a standard duty [92], while perceive such activity as unusual, outside their gender role and thus more stressful [93].

The absence of association between pain and childhood household dysfunction was perhaps surprising given past evidence on the negative impact of household dysfunction on chronic physical conditions [35,94]. Our result may reflect attenuation due to very high prevalence of these TEs in a population who grew up in during Apartheid in a ‘homeland’ with very limited income and public services [95]. As a result, our TE measures may not be precise enough to capture gradations of hardship. Alternatively, unmeasured history of chronic pain in families could generate residual confounding: chronic pain clusters within families so family history of pain increases the likelihood of children reporting the same issue [96], and familial chronic pain could increase child experience of TEs from, e.g., parental substance use, marital conflict or unemployment [17,97].

Exposure to war is unusually common in HAALSI, as one-third of the sample migrated from Mozambique due to civil war. However, our findings are congruent with Atwoli et al. [42] in not finding an association between war experience and chronic pain in South Africa. One possible reason for this lack of association may be selection bias–only the more resilient or least-affected individuals who had experienced war may have survived to be recruited into this study of older adults. This argument is somewhat supported by results from models stratified by country of origin: amongst South African origin respondents, war is non-significantly associated with more pain amongst South African origin respondents, but amongst Mozambiquan origin respondents where war experience is more common, the association is reversed.

The mechanisms between exposure to TEs and pain are unlikely to be identifiable in this cross-sectional study, and we therefore did not test for potential biological or psychological mediating processes proposed by existing literature. Future studies could use mediation analysis on longitudinal HAALSI data to investigate such hypotheses. Disentangling the etiology of pain, notably whether pain is more due to biological factors like diabetes or HIV neuropathy or psychological factors like PTSD or depression, would provide insight into appropriate approaches to pain prevention and treatment in this area.

Our study had some limitations. Although TEs and later life pain status have a clear temporal ordering, the data here were collected cross-sectionally, introducing the potential for reverse causation of TE reporting, if those experiencing pain now were more careful in recalling traumatic experiences. Path analysis, often used to emphasise causality in lifecourse research, was not perform due to the absence of temporal data on traumatic events. Social desirability bias for sensitive questions, such as sexual violence experiences, can lead to an underestimate TE prevalence, but the effect on associational estimates is unclear. More general recall failure may also occur when reporting on long-past events or those centred on others, something hinted at by the higher level of “don’t know” responses to TE questions on parental experiences. Unlike some other work, we could not differentiate between acute and chronic pain, since the Brief Pain Inventory evaluates severity rather than duration–although the question about ‘average pain’ intensity implicitly conveys chronicity. Since acute pain in Africa typically represents direct tissue injuries, such as trauma, labor, burns, or post-procedural pain [98], rather than the result of interactions between biopsychosocial factors [99,100], the association between earlier TEs and pain is more likely to reflect chronic pain than acute pain. Still, our results may contain additional imprecision if respondents were not always explicitly reporting chronic pain, indicating that further research is needed. As noted above, while similar questions relating to pain have been validated in this setting, the Brief Pain Inventory itself has not been. In-depth enquiry into cultural perceptions and manifestations of pain, and to formally validate pain measures in Shangaan and other South African Bantu languages, are important future steps. Finally, the HAALSI sample is from one rural area in western South Africa; the degree to which its results can be generalized to other settings should be carefully considered.

## Conclusions

Our findings suggest that the accumulation of TEs is associated with moderate-severe pain in later life among older rural South Africans, with notably strong associations for some specific TEs. Our work highlights the need for more research on TEs and pain in LMICs, given the high prevalence of both owing to unstable political and social contexts and aging populations, and the need for longitudinal follow-up to evaluate how TEs affect pain trajectories as well as point prevalence and causal mechanisms that might be amenable to later-life intervention to prevent chronic pain in older South African adults.

## Supporting information

S1 File SUPPORTING FIGURES S1-S2 AND SUPPORTING TABLES S1-S8. (DOCX)

## Abbreviations

HAALSI: Health and Aging in Africa: A longitudinal Study of an INDEPTH Community in South Africa
NCDs: Non-communicable diseases
YLDs: Years lived with disability
LMICs: Low and middle-income countries
TE: Traumatic event
PTSD: Post-traumatic stress disorder
HDSS: Health and socio-demographic surveillance system
ELSA: English Longitudinal Study of Aging
AIC: Akaike Information Criterion
CI: Confidence interval
SADHS: South African Demographic and Health Survey
SASH: South Africa Stress and Health

## References

[1] Raja SN, Carr DB, Cohen M, Finnerup NB, Flor H, Gibson S, et al. The revised International Association for the Study of Pain definition of pain: concepts, challenges, and compromises. Pain. 2020;161(9):1976–82. doi: 10.1097/j.pain.0000000000001939 32694387 PMC7680716

[2] Raffaeli W, Arnaudo E. Pain as a disease: an overview. Journal of Pain Research. 2017;Volume 10:2003–8. doi: 10.2147/JPR.S138864 28860855 PMC5573040

[3] Voscopoulos C, Lema M. When does acute pain become chronic? British Journal of Anaesthesia. 2010;105:i69–i85. doi: 10.1093/bja/aeq323 21148657

[4] Yong RJ, Mullins PM, Bhattacharyya N. Prevalence of chronic pain among adults in the United States. PAIN. 2022;163(2):e328–e32. doi: 10.1097/j.pain.0000000000002291 33990113

[5] Fayaz A, Croft P, Langford RM, Donaldson LJ, Jones GT. Prevalence of chronic pain in the UK: a systematic review and meta-analysis of population studies. BMJ Open. 2016;6(6):e010364. doi: 10.1136/bmjopen-2015-010364 27324708 PMC4932255

[6] Goldberg DS, McGee SJ. Pain as a global public health priority. BMC Public Health. 2011;11(1):770. 10.1186/1471-2458-11-770. 21978149 PMC3201926

[7] Magee D, Bachtold S, Brown M, Farquhar-Smith P. Cancer pain: where are we now? Pain Management. 2018;9(1):63–79. doi: 10.2217/pmt-2018-0031 30516438

[8] Miaskowski C, Blyth F, Nicosia F, Haan M, Keefe F, Smith A, et al. A Biopsychosocial Model of Chronic Pain for Older Adults. Pain Medicine. 2019. 10.1093/pm/pnz329. 31846035

[9] Institute for Health Metrics and Evaluation. Findings from the Global Burden of Disease Study 2017. Seattle, WA: IHME; 2018.

[10] Morriss WW, Roques CJ. Pain management in low- and middle-income countries. BJA Education. 2018;18(9):265–70. doi: 10.1016/j.bjae.2018.05.006 33456843 PMC7807826

[11] Parker R, Stein DJ, Jelsma J. Pain in people living with HIV/AIDS: a systematic review. Journal of the International AIDS Society. 2014;17(1):18719. doi: 10.7448/IAS.17.1.18719 24560338 PMC3929991

[12] Munsaka EF, Van Dyk D, Parker R. A retrospective audit of pain assessment and management post-caesarean section at New Somerset Hospital in Cape Town, South Africa. S Afr Fam Pract (2004). 2021;63(1):e1–e6. doi: 10.4102/safp.v63i1.5320 34636591 PMC8517764

[13] Wadley AL, Parker R, Mukhuba VA, Ratshinanga A, Zwane Z, Kamerman PR. South African men and women living with HIV have similar distributions of pain sites. Afr J Prim Health Care Fam Med. 2022;14(1):e1–e9. doi: 10.4102/phcfm.v14i1.3114 35144458 PMC8832001

[14] Bonezzi C, Fornasari D, Cricelli C, Magni A, Ventriglia G. Not All Pain is Created Equal: Basic Definitions and Diagnostic Work-Up. Pain Ther. 2020;9(Suppl 1):1–15. doi: 10.1007/s40122-020-00217-w 33315206 PMC7736598

[15] Gatchel RJ. Comorbidity of Chronic Pain and Mental Health Disorders: The Biopsychosocial Perspective. American Psychologist. 2004;59(8):795–805. doi: 10.1037/0003-066X.59.8.795 15554853

[16] Mills SEE, Nicolson KP, Smith BH. Chronic pain: a review of its epidemiology and associated factors in population-based studies. British Journal of Anaesthesia. 2019;123(2):e273–e83. doi: 10.1016/j.bja.2019.03.023 31079836 PMC6676152

[17] Nelson SM, Cunningham NR, Kashikar-Zuck S. A Conceptual Framework for Understanding the Role of Adverse Childhood Experiences in Pediatric Chronic Pain. Clin J Pain. 2017;33(3):264–70. doi: 10.1097/AJP.0000000000000397 27275737 PMC5143226

[18] Graf GH-J, Biroli P, Belsky DW. Critical Periods in Child Development and the Transition to Adulthood. JAMA Network Open. 2021;4(1):e2033359. doi: 10.1001/jamanetworkopen.2020.33359 33410874

[19] Payne CF, Mall S, Kobayashi L, Kahn K, Berkman L. Life-Course Trauma and Later Life Mental, Physical, and Cognitive Health in a Postapartheid South African Population: Findings From the HAALSI study. Journal of Aging and Health. 2020:089826432091345. 10.1177/0898264320913450. PMC8054553 32207348

[20] Lamvu G, Carrillo J, Ouyang C, Rapkin A. Chronic Pelvic Pain in Women. JAMA. 2021;325(23):2381. 10.1001/jama.2021.2631. 34128995

[21] Maccarrone J, Stripling A, Iannucci J, Nierenberg B. Exposure to trauma, PTSD and persistent pain in older adults: A systematic review. Aggression and Violent Behavior. 2021;57:101488. 10.1016/j.avb.2020.101488.

[22] Gasperi M, Afari N, Goldberg J, Suri P, Panizzon MS. Pain and Trauma: The Role of Criterion A Trauma and Stressful Life Events in the Pain and PTSD Relationship. The Journal of Pain. 2021;22(11):1506–17. doi: 10.1016/j.jpain.2021.04.015 34029685 PMC8578317

[23] Anda R, Tietjen G, Schulman E, Felitti V, Croft J. Adverse Childhood Experiences and Frequent Headaches in Adults. Headache: The Journal of Head and Face Pain. 2010;50(9):1473–81. doi: 10.1111/j.1526-4610.2010.01756.x 20958295

[24] England-Mason G, Casey R, Ferro M, MacMillan HL, Tonmyr L, Gonzalez A. Child maltreatment and adult multimorbidity: results from the Canadian Community Health Survey. Canadian Journal of Public Health. 2018;109(4):561–72. doi: 10.17269/s41997-018-0069-y 29981095 PMC6964466

[25] Scott KM. Association of Childhood Adversities and Early-Onset Mental Disorders With Adult-Onset Chronic Physical Conditions. Archives of General Psychiatry. 2011;68(8):838. doi: 10.1001/archgenpsychiatry.2011.77 21810647 PMC3402030

[26] Tan AC, Jaaniste T, Champion D. Chronic Widespread Pain and Fibromyalgia Syndrome: Life-Course Risk Markers in Young People. Pain Res Manag. 2019;2019:6584753–. doi: 10.1155/2019/6584753 31191788 PMC6525804

[27] Jones GT, Power C, Macfarlane GJ. Adverse events in childhood and chronic widespread pain in adult life: Results from the 1958 British Birth Cohort Study. Pain. 2009;143(1–2):92–6. doi: 10.1016/j.pain.2009.02.003 19304391

[28] Baumeister D, Akhtar R, Ciufolini S, Pariante CM, Mondelli V. Childhood trauma and adulthood inflammation: a meta-analysis of peripheral C-reactive protein, interleukin-6 and tumour necrosis factor-α. Molecular Psychiatry. 2016;21(5):642–9. 10.1038/mp.2015.67. 26033244 PMC4564950

[29] Lacey RE, Pinto Pereira SM, Li L, Danese A. Adverse childhood experiences and adult inflammation: Single adversity, cumulative risk and latent class approaches. Brain, Behavior, and Immunity. 2020. doi: 10.1016/j.bbi.2020.03.017 32201253 PMC7327510

[30] Bush NR, Lane RD, McLaughlin KA. Mechanisms Underlying the Association Between Early-Life Adversity and Physical Health. Psychosomatic Medicine. 2016;78(9):1114–9. 10.1097/psy.0000000000000421. 27763991 PMC5111624

[31] McLaughlin KA, Basu A, Walsh K, Slopen N, Sumner JA, Koenen KC, et al. Childhood exposure to violence and chronic physical conditions in a national sample of US adolescents. Psychosomatic Medicine. 2016;78(9):1072–83. doi: 10.1097/PSY.0000000000000366 27428855 PMC5096968

[32] You DS, Meagher MW. Childhood Adversity and Pain Sensitization. Psychosom Med. 2016;78(9):1084–93. doi: 10.1097/PSY.0000000000000399 27755280

[33] Nicol AL, Sieberg CB, Clauw DJ, Hassett AL, Moser SE, Brummett CM. The Association Between a History of Lifetime Traumatic Events and Pain Severity, Physical Function, and Affective Distress in Patients With Chronic Pain. J Pain. 2016;17(12):1334–48. doi: 10.1016/j.jpain.2016.09.003 27641311

[34] Hiscox LV, Hiller R, Fraser A, Rabie S, Stewart J, Seedat S, et al. Sex differences in post-traumatic stress disorder in a high adversity cohort of South African adolescents: an examination of depressive symptoms, age, and trauma type as explanatory factors. European Journal of Psychotraumatology. 2021;12(1). 10.1080/20008198.2021.1978669. PMC8530480 34691370

[35] Ramiro LS, Madrid BJ, Brown DW. Adverse childhood experiences (ACE) and health-risk behaviors among adults in a developing country setting. Child Abuse Negl. 2010;34(11):842–55. doi: 10.1016/j.chiabu.2010.02.012 20888640

[36] Flores-Torres MH, Comerford E, Signorello L, Grodstein F, Lopez-Ridaura R, de Castro F, et al. Impact of adverse childhood experiences on cardiovascular disease risk factors in adulthood among Mexican women. Child Abuse Negl. 2020;99:104175. doi: 10.1016/j.chiabu.2019.104175 31710961

[37] Kidman R, Piccolo LR, Kohler H-P. Adverse Childhood Experiences: Prevalence and Association With Adolescent Health in Malawi. American Journal of Preventive Medicine. 2020;58(2):285–93. doi: 10.1016/j.amepre.2019.08.028 31810632 PMC6981018

[38] Jordans MJD, Pigott H, Tol WA. Interventions for Children Affected by Armed Conflict: a Systematic Review of Mental Health and Psychosocial Support in Low- and Middle-Income Countries. Current Psychiatry Reports. 2016;18(1). doi: 10.1007/s11920-015-0648-z 26769198 PMC4713453

[39] Miranda JJ, Kinra S, Casas JP, Davey Smith G, Ebrahim S. Non-communicable diseases in low- and middle-income countries: context, determinants and health policy. Trop Med Int Health. 2008;13(10):1225–34. doi: 10.1111/j.1365-3156.2008.02116.x 18937743 PMC2687091

[40] Jacob C, Baird J, Barker M, Cooper C, Hanson M. The importance of a life course approach to health: chronic disease risk from preconception through adolescence and adulthood. Southampton: University of Southampton. 2015. https://eprints.soton.ac.uk/436656/.

[41] Manyema M, Richter LM. Adverse childhood experiences: prevalence and associated factors among South African young adults. Heliyon. 2019;5(12):e03003. doi: 10.1016/j.heliyon.2019.e03003 31890957 PMC6926197

[42] Atwoli L, Platt JM, Basu A, Williams DR, Stein DJ, Koenen KC. Associations between lifetime potentially traumatic events and chronic physical conditions in the South African Stress and Health Survey: a cross-sectional study. Bmc Psychiatry. 2016;16. doi: 10.1186/s12888-016-0929-z 27389090 PMC4936266

[43] Kamerman PR, Bradshaw D, Laubscher R, Pillay-Van Wyk V, Gray GE, Mitchell D, et al. Almost 1 in 5 South African adults have chronic pain: a prevalence study conducted in a large nationally representative sample. Pain. 2020;161(7):1629–35. doi: 10.1097/j.pain.0000000000001844 32102020

[44] Sideris T. War, gender and culture: Mozambican women refugees. Social Science & Medicine. 2003;56(4):713–24. doi: 10.1016/s0277-9536(02)00067-9 12560006

[45] Gómez-Olivé FX, Montana L, Wagner RG, Kabudula CW, Rohr JK, Kahn K, et al. Cohort Profile: Health and Ageing in Africa: A Longitudinal Study of an INDEPTH Community in South Africa (HAALSI). International Journal of Epidemiology. 2018;47(3):689–90j. doi: 10.1093/ije/dyx247 29325152 PMC6005147

[46] Williams J, Ibisomi L, Sartorius B, Kahn K, Collinson M, Tollman S, et al. Convergence in fertility of South Africans and Mozambicans in rural South Africa, 1993–2009. Glob Health Action. 2013;6:19236–. doi: 10.3402/gha.v6i0.19236 23364078 PMC3556705

[47] Cleeland CS, Ryan K. The brief pain inventory. Pain Research Group. 1991:143–7.

[48] Daut RL, Cleeland CS, Flanery RC. Development of the Wisconsin Brief Pain Questionnaire to assess pain in cancer and other diseases. Pain. 1983;17(2):197–210. doi: 10.1016/0304-3959(83)90143-4 6646795

[49] Mphahlele N, Mitchell D, Kamerman P. Validation of the Wisconsin Brief Pain Questionnaire in a Multilingual South African Population. Journal of Pain and Symptom Management. 2008;36(4):396–412. doi: 10.1016/j.jpainsymman.2007.10.020 18448308

[50] Kawai K, Kawai AT, Wollan P, Yawn BP. Adverse impacts of chronic pain on health-related quality of life, work productivity, depression and anxiety in a community-based study. Family Practice. 2017;34(6):656–61. doi: 10.1093/fampra/cmx034 28444208 PMC6260800

[51] Tan G, Jensen MP, Thornby JI, Rintala DH, Anderson KO. Categorizing pain in patients seen in a veterans health administration hospital: Pain as the fifth vital sign. Psychological Services. 2008;5(3):239–50. 10.1037/1541-1559.5.3.239.

[52] Miró J, de la Vega R, Solé E, Racine M, Jensen MP, Gálan S, et al. Defining mild, moderate, and severe pain in young people with physical disabilities. Disabil Rehabil. 2017;39(11):1131–5. doi: 10.1080/09638288.2016.1185469 27291566 PMC5553452

[53] Zelman DC, Dukes E, Brandenburg N, Bostrom A, Gore M. Identification of cut-points for mild, moderate and severe pain due to diabetic peripheral neuropathy. Pain. 2005;115(1):29–36. doi: 10.1016/j.pain.2005.01.028 15836967

[54] Steptoe A, Breeze E, Banks J, Nazroo J. Cohort profile: the English longitudinal study of ageing. Int J Epidemiol. 2013;42(6):1640–8. doi: 10.1093/ije/dys168 23143611 PMC3900867

[55] Kessler RC, Aguilar-Gaxiola S, Alonso J, Benjet C, Bromet EJ, Cardoso G, et al. Trauma and PTSD in the WHO World Mental Health Surveys. European Journal of Psychotraumatology. 2017;8(sup5):1353383. doi: 10.1080/20008198.2017.1353383 29075426 PMC5632781

[56] Liu H, Petukhova MV, Sampson NA, Aguilar-Gaxiola S, Alonso J, Andrade LH, et al. Association of DSM-IV Posttraumatic Stress Disorder With Traumatic Experience Type and History in the World Health Organization World Mental Health Surveys. JAMA Psychiatry. 2017;74(3):270. doi: 10.1001/jamapsychiatry.2016.3783 28055082 PMC5441566

[57] Chartier MJ, Walker JR, Naimark B. Separate and cumulative effects of adverse childhood experiences in predicting adult health and health care utilization. Child Abuse Negl. 2010;34(6):454–64. doi: 10.1016/j.chiabu.2009.09.020 20409586

[58] Fonseka RW, Minnis AM, Gomez AM. Impact of Adverse Childhood Experiences on Intimate Partner Violence Perpetration among Sri Lankan Men. PLOS ONE. 2015;10(8):e0136321. doi: 10.1371/journal.pone.0136321 26295577 PMC4546656

[59] Bartley EJ, Fillingim RB. Sex differences in pain: a brief review of clinical and experimental findings. Br J Anaesth. 2013;111(1):52–8. doi: 10.1093/bja/aet127 23794645 PMC3690315

[60] Erola J, Jalonen S, Lehti H. Parental education, class and income over early life course and children’s achievement. Research in Social Stratification and Mobility. 2016;44:33–43. 10.1016/j.rssm.2016.01.003.

[61] Lien N. Adolescents’ proxy reports of parents’ socioeconomic status: How valid are they? Journal of Epidemiology & Community Health. 2001;55(10):731–7. 10.1136/jech.55.10.731. 11553657 PMC1731778

[62] International Labour Organization. International Standard Classification of Occupations 2008 (ISCO-08): Structure, group definitions and correspondence tables. Geneva: 2012.

[63] Kobayashi L, Glymour M, Kahn K, Payne CF, Wagner R, Montana L, et al. Childhood deprivation and later-life cognitive function in a population-based study of older rural South Africans. Social Science & Medicine. 2017;190:20–8. 10.1016/j.socscimed.2017.08.009. 28837862 PMC5915343

[64] R Core Team. R: A language and environment for statistical computing. R Foundation for Statistical Computing, Vienna, Austria. 2019. https://www.R-project.org/.

[65] Häuser W, Glaesmer H, Schmutzer G, Brähler E. Widespread pain in older Germans is associated with posttraumatic stress disorder and lifetime employment status–Results of a cross-sectional survey with a representative population sample. PAIN®. 2012;153(12):2466–72. doi: 10.1016/j.pain.2012.09.006 23084003

[66] Loeb TB, Joseph NT, Wyatt GE, Zhang M, Chin D, Thames A, et al. Predictors of somatic symptom severity: The role of cumulative history of trauma and adversity in a diverse community sample. Psychological Trauma: Theory, Research, Practice, and Policy. 2018;10(5):491. doi: 10.1037/tra0000334 29154595 PMC6021222

[67] Ben-Shlomo Y, Cooper R, Kuh D. The last two decades of life course epidemiology, and its relevance for research on ageing. International Journal of Epidemiology. 2016;45(4):973–88. doi: 10.1093/ije/dyw096 27880685 PMC5841628

[68] Kuh D. Life course epidemiology. Journal of Epidemiology & Community Health. 2003;57(10):778–83. doi: 10.1136/jech.57.10.778 14573579 PMC1732305

[69] Baldwin JR, Arseneault L, Caspi A, Fisher HL, Moffitt TE, Odgers CL, et al. Childhood victimization and inflammation in young adulthood: A genetically sensitive cohort study. Brain, behavior, and immunity. 2018;67:211–7. doi: 10.1016/j.bbi.2017.08.025 28867281 PMC5710993

[70] Ogle CM, Rubin DC, Siegler IC. Cumulative exposure to traumatic events in older adults. Aging & Mental Health. 2014;18(3):316–25. doi: 10.1080/13607863.2013.832730 24011223 PMC3944195

[71] Halpern CT, Tucker CM, Bengtson A, Kupper LL, McLean SA, Martin SL. Somatic symptoms among US adolescent females: associations with sexual and physical violence exposure. Matern Child Health J. 2013;17(10):1951–60. doi: 10.1007/s10995-013-1221-1 23340952 PMC3689845

[72] Sansone RA, Watts DA, Wiederman MW. Childhood trauma and pain and pain catastrophizing in adulthood: a cross-sectional survey study. Prim Care Companion CNS Disord. 2013;15(4):PCC.13m01506. doi: 10.4088/PCC.13m01506 24392263 PMC3869615

[73] Wuest J, O’Donnell S, Scott-Storey K, Malcolm J, Vincent CD, Taylor P. Cumulative Lifetime Violence Severity and Chronic Pain in a Community Sample of Canadian Men. Pain Medicine. 2021;22(6):1387–98. doi: 10.1093/pm/pnaa419 33347593

[74] Paras ML, Murad MH, Chen LP, Goranson EN, Sattler AL, Colbenson KM, et al. Sexual Abuse and Lifetime Diagnosis of Somatic Disorders. JAMA. 2009;302(5):550. 10.1001/jama.2009.1091. 19654389

[75] Filiatreau LM, Giovenco D, Twine R, Gómez‐Olivé FX, Kahn K, Haberland N, et al. Examining the relationship between physical and sexual violence and psychosocial health in young people living with HIV in rural South Africa. Journal of the International AIDS Society. 2020;23(12). 10.1002/jia2.25654. PMC7749553 33340267

[76] Kaminer D, Grimsrud A, Myer L, Stein DJ, Williams DR. Risk for post-traumatic stress disorder associated with different forms of interpersonal violence in South Africa. Social Science & Medicine. 2008;67(10):1589–95. doi: 10.1016/j.socscimed.2008.07.023 18774211 PMC2610682

[77] Treves-Kagan S, Maman S, Khoza N, Macphail C, Peacock D, Twine R, et al. Fostering gender equality and alternatives to violence: perspectives on a gender-transformative community mobilisation programme in rural South Africa. Culture, Health & Sexuality. 2020;22(sup1):127–44. doi: 10.1080/13691058.2019.1650397 31429663 PMC7905832

[78] Angeletti C, Guetti C, Ursini ML, Taylor R, Papola R, Petrucci E, et al. Low Back Pain in a Natural Disaster. Pain Practice. 2014;14(2):E8–E16. doi: 10.1111/papr.12087 23763663

[79] Arcaya MC, Lowe SR, Asad AL, Subramanian SV, Waters MC, Rhodes J. Association of posttraumatic stress disorder symptoms with migraine and headache after a natural disaster. Health Psychol. 2017;36(5):411–8. doi: 10.1037/hea0000433 27929328 PMC6666314

[80] Beaglehole B, Mulder RT, Frampton CM, Boden JM, Newton-Howes G, Bell CJ. Psychological distress and psychiatric disorder after natural disasters: systematic review and meta-analysis. The British Journal of Psychiatry. 2018;213(6):716–22. doi: 10.1192/bjp.2018.210 30301477

[81] Raker EJ, Arcaya MC, Lowe SR, Zacher M, Rhodes J, Waters MC. Mitigating Health Disparities After Natural Disasters: Lessons From The RISK Project: Study examines mitigating health disparities after natural disasters. Health Affairs. 2020;39(12):2128–35. 10.1377/hlthaff.2020.01161. 33284697 PMC8533028

[82] Sekiguchi T, Hagiwara Y, Sugawara Y, Tomata Y, Tanji F, Watanabe T, et al. Influence of subjective economic hardship on new onset of neck pain (so-called: katakori) in the chronic phase of the Great East Japan Earthquake: A prospective cohort study. J Orthop Sci. 2018;23(5):758–64. doi: 10.1016/j.jos.2018.04.011 29933941

[83] Kılıc C, Aydın İ, Taşkıntuna N, Ozcurumez G, Kurt G, Eren E, et al. Predictors of psychological distress in survivors of the 1999 earthquakes in Turkey: effects of relocation after the disaster. Acta Psychiatrica Scandinavica. 2006;114(3):194–202. doi: 10.1111/j.1600-0447.2006.00786.x 16889590

[84] McEniry M, Samper-Ternent R, Cano-Gutierrez C. Displacement due to armed conflict and violence in childhood and adulthood and its effects on older adult health: The case of the middle-income country of Colombia. SSM—Population Health. 2019;7. doi: 10.1016/j.ssmph.2019.100369 30859118 PMC6396198

[85] Holley AL, Wilson AC, Noel M, Palermo TM. Post-traumatic stress symptoms in children and adolescents with chronic pain: A topical review of the literature and a proposed framework for future research. Eur J Pain. 2016;20(9):1371–83. doi: 10.1002/ejp.879 27275585 PMC5912261

[86] Hiskey S, Luckie M, Davies S, Brewin CR. The Emergence of Posttraumatic Distress in Later Life: A Review. Journal of Geriatric Psychiatry and Neurology. 2008;21(4):232–41. doi: 10.1177/0891988708324937 19017780

[87] Jones SL, Hadjistavropoulos HD, Janzen JA, Hadjistavropoulos T. The Relation of Pain and Caregiver Burden in Informal Older Adult Caregivers. Pain Medicine. 2011;12(1):51–8. doi: 10.1111/j.1526-4637.2010.01018.x 21143758

[88] Kuo C, Operario D. Health of adults caring for orphaned children in an HIV-endemic community in South Africa. AIDS Care. 2011;23(9):1128–35. doi: 10.1080/09540121.2011.554527 21480009 PMC3139727

[89] Kidman R, Thurman TR. Caregiver burden among adults caring for orphaned children in rural South Africa. Vulnerable Children and Youth Studies. 2014;9(3):234–46. doi: 10.1080/17450128.2013.871379 24999368 PMC4066884

[90] Casale M. ‘I am living a peaceful life with my grandchildren. Nothing else.’ Stories of adversity and ‘resilience’ of older women caring for children in the context of HIV/AIDS and other stressors. Ageing and Society. 2011;31(8):1265–88. 10.1017/s0144686x10001303.

[91] Pinquart M, Sorensen S. Gender Differences in Caregiver Stressors, Social Resources, and Health: An Updated Meta-Analysis. The Journals of Gerontology Series B: Psychological Sciences and Social Sciences. 2006;61(1):P33–P45. doi: 10.1093/geronb/61.1.p33 16399940

[92] Schatz EJ. “Taking care of my own blood’’: Older women’s relationships to their households in rural South Africa. Scandinavian Journal of Public Health. 2007;35(69_suppl):147–54. doi: 10.1080/14034950701355676 17676516 PMC2830102

[93] Schatz E, Seeley J. Gender, ageing and carework in East and Southern Africa: A review. Global Public Health. 2015;10(10):1185–200. doi: 10.1080/17441692.2015.1035664 25947225 PMC4888771

[94] Felitti VJ, Anda RF, Nordenberg D, Williamson DF, Spitz AM, Edwards V, et al. Relationship of Childhood Abuse and Household Dysfunction to Many of the Leading Causes of Death in Adults. American Journal of Preventive Medicine. 1998;14(4):245–58. 10.1016/s0749-3797(98)00017-8. 9635069

[95] Collinson MA, White MJ, Bocquier P, McGarvey ST, Afolabi SA, Clark SJ, et al. Migration and the epidemiological transition: insights from the Agincourt sub-district of northeast South Africa. Glob Health Action. 2014;7(1):23514. doi: 10.3402/gha.v7.23514 24848656 PMC4028907

[96] Van Hecke O, Torrance N, Smith BH. Chronic pain epidemiology and its clinical relevance. British Journal of Anaesthesia. 2013;111(1):13–8. doi: 10.1093/bja/aet123 23794640

[97] Snelling J. The effect of chronic pain on the family unit. Journal of Advanced Nursing. 1994;19(3):543–51. doi: 10.1111/j.1365-2648.1994.tb01119.x 8014316

[98] Lourens A, Mccaul M, Parker R, Hodkinson P. Acute Pain in the African Prehospital Setting: A Scoping Review. Pain Research and Management. 2019;2019:1–13. doi: 10.1155/2019/2304507 31149317 PMC6501243

[99] Delaney LD, Clauw DJ, Waljee JF. The Management of Acute Pain for Musculoskeletal Conditions: The Challenges of Opioids and Opportunities for the Future. J Bone Joint Surg Am. 2020;102(Suppl 1):3–9. doi: 10.2106/JBJS.20.00228 32251126 PMC8272973

[100] Ekman EF, Koman LA. Acute pain following musculoskeletal injuries and orthopaedic surgery: mechanisms and management. Instr Course Lect. 2005;54:21–33. http://europepmc.org/abstract/MED/15948432. 15948432

