# Supplementary material for: The relationship between lifecourse traumatic events and pain in an older rural South African population: A cross-sectional study
Source: PLoS One. 2024 Dec 16;19(12):e0313140. doi: 10.1371/journal.pone.0313140 (PMC11649084; doi:10.1371/journal.pone.0313140)
Supplement: S1 File — (DOCX) [file pone.0313140.s001.docx]

# Supplementary Material

**Title**: The relationship between lifecourse traumatic events and pain in a rural South African population: a cross-sectional study

S1 Figure. Directed acyclic graph between TEs, pain, and other covariates


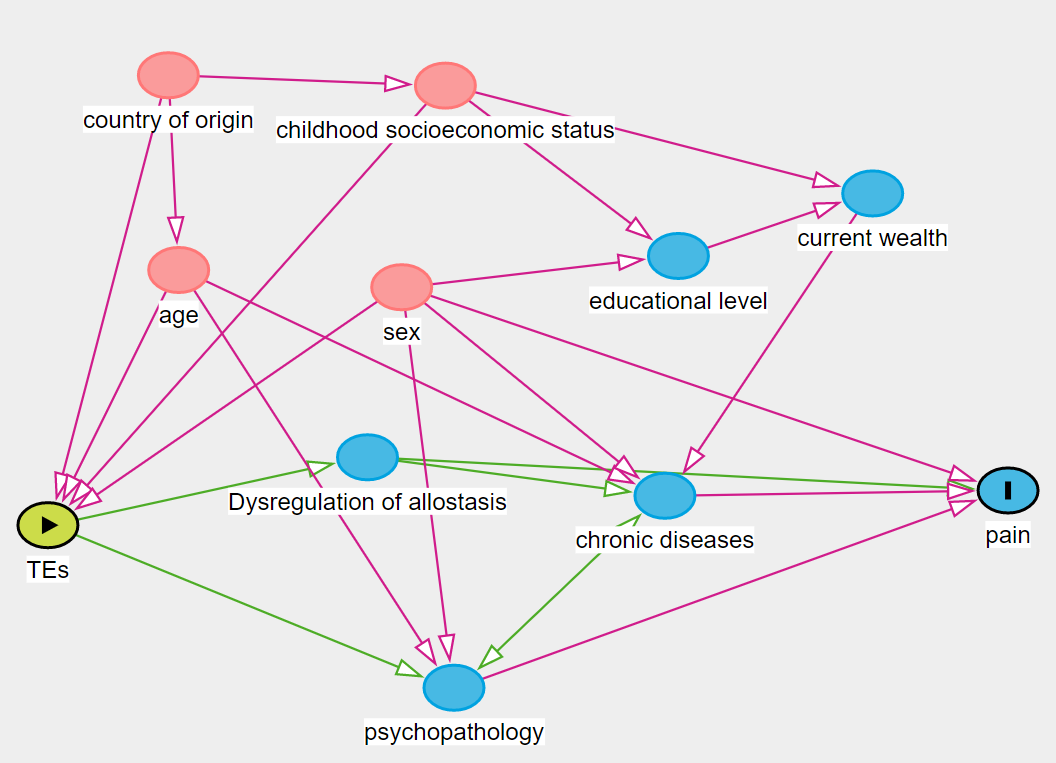


S2 Figure. Comparing cumulative TE count model and quadratic TE count model on AIC and predicted probabilities plots

|  | **Cumulative model** | **Quadratic model** |
| --- | --- | --- |
| **AIC** | 1289.1 | 1286.3 |
| **Predicted  probability  plots** | **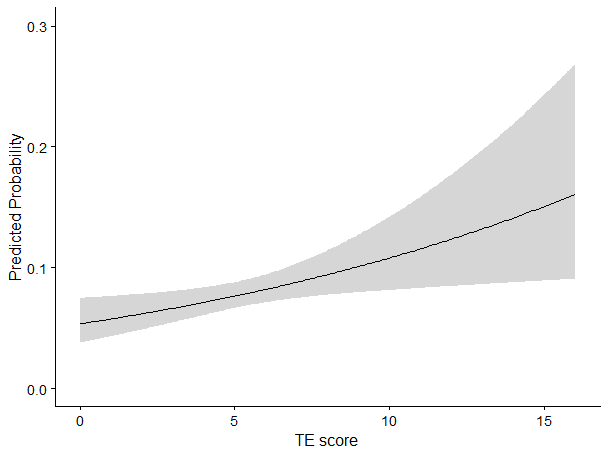** | **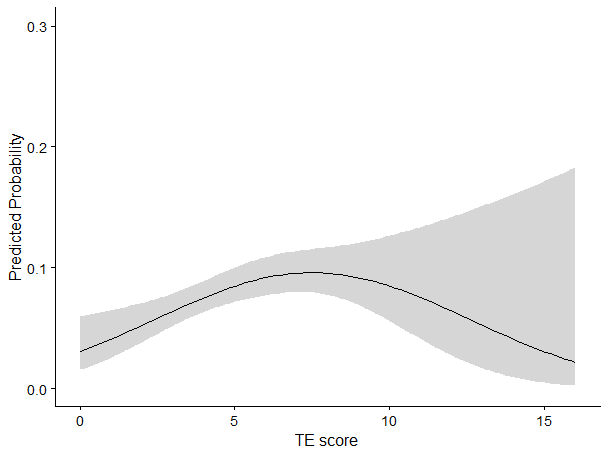** |

*Note: AIC = Akaike Information Criterion*

S1 Table. Multivariable ordinal logistic regressions of mild, moderate and severe pain (Proportional odds ratios and 95% confidence intervals)

|  | **TE score** | |  | **TE categories** | | | | | |
| --- | --- | --- | --- | --- | --- | --- | --- | --- | --- |
|  | **Bivariate** | **Multivariable** |  | **Bivariate** | **Multivariable** | | | | |
| **Traumatic event count** | 1.08  (1.02-1.14) | 1.10  (1.03-1.16) |  |  |  |  |  |  |  |
| **Childhood household dysfunction** |  |  |  | 0.95  (0.72-1.26) | 1.04  (0.78-1.39) |  |  |  |  |
| **Social/family environment** |  |  |  | 1.06  (0.70-1.68) |  | 1.09  (0.71-1.73) |  |  |  |
| **Violence in the community** |  |  |  | 1.38  (1.03-1.87) |  |  | 1.51  (1.11-2.06) |  |  |
| **Natural disaster, illness and accident** |  |  |  | 1.66  (1.03-2.82) |  |  |  | 1.80  (1.10-3.12) |  |
| **War related** |  |  |  | 1.05  (0.75-1.45) |  |  |  |  | 1.16  (0.80-1.66) |
| **Age** |  |  |  |  |  |  |  |  |  |
| 40-49 |  | Ref. |  |  | Ref. | Ref. | Ref. | Ref. | Ref. |
| 50-59 |  | 1.99  (1.28-3.2) |  |  | 2.07  (1.34-3.29) | 2.06  (1.33-3.28) | 2.06  (1.33-3.27) | 2.03  (1.31-3.24) | 2.05  (1.33-3.26) |
| 60-69 |  | 2.26  (1.41-3.69) |  |  | 2.34 (1.46-3.84) | 2.32  (1.45-3.78) | 2.25 (1.40-3.67) | 2.22  (1.38-3.63) | 2.32  (1.4-3.78) |
| 70-79 |  | 2.75  (1.74-4.47) |  |  | 2.71  (1.71-4.39) | 2.71 (1.71-4.40) | 2.80 (1.76-4.55) | 2.94 (1.84-4.79) | 2.68  (1.69-4.35) |
| **Sex** |  |  |  |  |  |  |  |  |  |
| Female vs male |  | 1.72  (1.27-2.34) |  |  | 1.66  (1.23-2.25) | 1.65  (1.23-2.25) | 1.74  (1.29-2.37) | 1.64  (1.22-2.22) | 1.68  (1.24-2.28) |
| **Country of origin** |  |  |  |  |  |  |  |  |  |
| Mozambique/other vs South Africa |  | 0.85  (0.61-1.17) |  |  | 0.92  (0.66-1.25) | 0.91  (0.66-1.25) | 0.90  (0.65-1.23) | 0.90 (0.65-1.23) | 0.87  (0.61-1.22) |
| **Father’s occupation** |  |  |  |  |  |  |  |  |  |
| Skilled vs unskilled |  | 1.02  (0.73-1.45) |  |  | 1.04  (0.74-1.47) | 1.04  (0.46-1.39) | 1.03  (0.73-1.45) | 1.04 (0.74-1.46) | 0.81  (0.45-1.39) |

*Note: Results are from twelve separate ordinal logistic regressions. Reference group in all cases is the combination of ‘did not experience any TE in the category’ and those with ‘non-affirmative’ answer in the TE category. Multivariable models were adjusted for age, sex, country of origin and father’s occupation. POR= proportional odds ratio; APOR: adjusted proportional odds ratio; CI= confidence interval*

S2 Table. Logistic regressions of moderate and severe pain with exposure to TE score and TE categories stratified by country of origin

|  | **Multivariable analysis** | | |
| --- | --- | --- | --- |
|  | **AOR (95% CI)** | | |
|  | **Total (N=2411)** | **South Africa (N=1721)** | **Mozambique/other (N=690)** |
| **Traumatic event count** | 1.09 (1.03-1.16) | 1.12 (1.05-1.21) | 1.01 (0.91-1.13) |
|  |  |  |  |
| **Childhood household dysfunction** | 1.00 (0.73-1.36) | 1.07 (0.75-1.56) | 0.79 (0.44-1.42) |
| **Social/family environment** | 1.18 (0.74-1.97) | 1.19 (0.70-2.17) | 1.05 (0.43-3.15) |
| **Violence in the community** | 1.62 (1.17-2.27) | 1.80 (1.22-2.72) | 1.28 (0.71-2.41) |
| **Natural disaster, illness and accident** | 1.70 (1.01-3.05) | 1.76 (0.96-3.50) | 1.53 (0.58-5.27) |
| **War-related** | 1.06 (0.71-1.57) | 1.52 (0.91-2.47) | 0.71 (0.39-1.25) |

Note: Results are from eighteen separate logistic regressions. Reference group in all cases is the combination of ‘did not experience any TE in the category’ and those with ‘any don’t know’ answers in that TE category. Multivariable models were adjusted for age, sex, country of origin and father’s occupation, except when stratified on country of origin. OR= odds ratio; AOR: adjusted odds ratio; CI= confidence interval.

S3 Table. Bivariate and multivariable logistic regressions of moderate and severe pain across covariates

|  | **Bivariate analysis** | **Multivariable analysis** | | |
| --- | --- | --- | --- | --- |
|  | **OR (95% CI)** | **AOR (95% CI)** | | |
|  |  | **Total** | **Female** | **Male** |
| **Age** |  |  |  |  |
| 40-49 | Ref. | Ref. | Ref. | Ref. |
| 50-59 | 1.89(1.20-3.07) | 1.79 (1.13-2.91) | 1.87 (1.08-3.37) | 1.59 (0.70-3.96) |
| 60-69 | 2.12 (1.30-3.51) | 2.19 (1.33-3.65) | 2.62 (1.46-4.86) | 1.44 (0.58-3.76) |
| 70-79 | 2.29 (1.41-3.79) | 2.47 (1.52-4.12) | 2.24 (1.22-4.24) | 2.70 (1.20-6.66) |
| **Sex** |  |  |  |  |
| Female vs male | 1.64 (1.19-2.27) | 1.79 (1.30-2.49) |  |  |
| **Country of origin** |  |  |  |  |
| Mozambique/other vs South Africa | 0.96 (0.68-1.33) | 0.90 (0.63-1.25) | 0.89 (0.58-1.33) | 0.88 (0.46-1.61) |
| **Father’s occupation** |  |  |  |  |
| Skilled vs unskilled | 1.09 (0.76-1.58) | 1.13 (0.78-1.65) | 1.00 (0.65-1.55) | 1.64 (0.80-3.72) |

*Note: OR= odds ratio; AOR: adjusted odds ratio; CI= confidence interval*

S4 Table. Result of multiple logistic regressions of moderate and severe pain across covariates (with the exposure to TE category: childhood household dysfunction)

|  | **Multivariable analysis** | | |
| --- | --- | --- | --- |
|  | **AOR (95% CI)** | | |
|  | **Total** | **Female** | **Male** |
| **Childhood household dysfunction** | 1.00 (0.73-1.36) | 0.91 (0.63-1.32) | 1.26 (0.72-2.28) |
| **Age** |  |  |  |
| 40-49 | Ref. | Ref. | Ref. |
| 50-59 | 1.86 (1.17-3.02) | 1.93 (1.12-3.47) | 1.68 (0.74-4.17) |
| 60-69 | 2.25 (1.37-3.77) | 2.67 (1.48-4.95) | 1.57 (0.63-4.16) |
| 70-79 | 2.42 (1.49-4.03) | 2.25 (1.22-4.25) | 2.67 (1.18-6.62) |
| **Sex** |  |  |  |
| Female vs male | 1.73 (1.25-2.40) |  |  |
| **Country of origin** |  |  |  |
| Mozambique/other vs South Africa | 0.96 (0.68-1.33) | 0.93 (0.61-1.39) | 0.96 (0.51-1.73) |
| **Father’s occupation** |  |  |  |
| Skilled vs unskilled | 1.15 (0.80-1.68) | 1.01 (0.66-1.57) | 1.71 (0.83-3.88) |

*Note: Results are from multiple logistic regressions. The multivariable analysis was adjusted for age, sex, country of origin and father’s occupation. Sex was stratified in other two multivariable analyses. OR= odds ratio; AOR: adjusted odds ratio; CI= confidence interval*

S5 Table. Result of multiple logistic regressions of moderate and severe pain across covariates (with the exposure to TE category: social/family environment)

|  | **Multivariable analysis** | | |
| --- | --- | --- | --- |
|  | **AOR (95% CI)** | | |
|  | **Total** | **Female** | **Male** |
| **Social/family environment** | 1.18 (0.74-1.97) | 0.97 (0.56-1.78) | 1.80 (0.77-5.31) |
| **Age** |  |  |  |
| 40-49 | Ref. | Ref. | Ref. |
| 50-59 | 1.85 (1.17-3.01) | 1.94 (1.12-3.49) | 1.63 (0.71-4.05) |
| 60-69 | 2.24 (1.37-3.74) | 2.72 (1.52-5.03) | 1.44 (0.58-3.77) |
| 70-79 | 2.44 (1.50-4.06) | 2.24 (1.22-4.24) | 2.58 (1.15-6.36) |
| **Sex** |  |  |  |
| Female vs male | 1.72 (1.25-2.40) |  |  |
| **Country of origin** |  |  |  |
| Mozambique/other vs South Africa | 0.96 (0.68-1.33) | 0.94 (0.62-1.40) | 0.95 (0.51-1.71) |
| **Father’s occupation** |  |  |  |
| Skilled vs unskilled | 1.15 (0.80-1.68) | 1.01 (0.66-1.57) | 1.67 (0.81-3.78) |

*Note: Results are from multiple logistic regressions. The multivariable analysis was adjusted for age, sex, country of origin and father’s occupation. Sex was stratified in other two multivariable analyses. OR= odds ratio; AOR: adjusted odds ratio; CI= confidence interval*

S6 Table. Result of multiple logistic regressions of moderate and severe pain across covariates (with the exposure to TE category: violence in the community)

|  | **Multivariable analysis** | | |
| --- | --- | --- | --- |
|  | **AOR (95% CI)** | | |
|  | **Total** | **Female** | **Male** |
| **Violence in the community** | 1.62 (1.17-2.27) | 1.93 (1.30-2.90) | 1.14 (0.64-2.12) |
| **Age** |  |  |  |
| 40-49 | Ref. | Ref. | Ref. |
| 50-59 | 1.84 (1.16-3.00) | 1.92 (1.11-3.45) | 1.65 (0.72-4.11) |
| 60-69 | 2.17 (1.33-3.62) | 2.59 (1.44-4.81) | 1.44 (0.58-3.77) |
| 70-79 | 2.52 (1.55-4.20) | 2.28 (1.24-4.32) | 2.60 (1.15-6.44) |
| **Sex** |  |  |  |
| Female vs male | 1.83 (1.33-2.55) |  |  |
| **Country of origin** |  |  |  |
| Mozambique/other vs South Africa | 0.94 (0.66-1.31) | 0.90 (0.59-1.35) | 0.97 (0.51-1.74) |
| **Father’s occupation** |  |  |  |
| Skilled vs unskilled | 1.13 (0.79-1.65) | 0.97 (0.63-1.50) | 1.71 (0.83-3.86) |

*Note: Results are from multiple logistic regressions. The multivariable analysis was adjusted for age, sex, country of origin and father’s occupation. Sex was stratified in other two multivariable analyses. OR= odds ratio; AOR: adjusted odds ratio; CI= confidence interval*

S7 Table. Result of multiple logistic regressions of moderate and severe pain across covariates (with the exposure to TE category: natural disaster, illness and accident)

|  | **Multivariable analysis** | | |
| --- | --- | --- | --- |
|  | **AOR (95% CI)** | | |
|  | **Total** | **Female** | **Male** |
| **Natural disaster, illness and accident** | 1.70 (1.01-3.05) | 1.81 (0.93-3.98) | 1.56 (0.70-4.00) |
| **Age** |  |  |  |
| 40-49 | Ref. | Ref. | Ref. |
| 50-59 | 1.83 (1.16-2.98) | 1.91 (1.10-3.43) | 1.64 (0.72-4.08) |
| 60-69 | 2.16 (1.32-3.61) | 2.58 (1.44-4.78) | 1.43 (0.58-3.72) |
| 70-79 | 2.62 (1.60-4.38) | 2.41 (1.30-4.57) | 2.77 (1.22-6.91) |
| **Sex** |  |  |  |
| Female vs male | 1.71 (1.24-2.38) |  |  |
| **Country of origin** |  |  |  |
| Mozambique/other vs South Africa | 0.94 (0.67-1.32) | 0.93 (0.61-1.39) | 0.94 (0.50-1.70) |
| **Father’s occupation** |  |  |  |
| Skilled vs unskilled | 1.14 (0.80-1.67) | 1.00 (0.65-1.56) | 1.69 (0.82-3.82) |

*Note: Results are from multiple logistic regressions. The multivariable analysis was adjusted for age, sex, country of origin and father’s occupation. Sex was stratified in other two multivariable analyses. OR= odds ratio; AOR: adjusted odds ratio; CI= confidence interval*

S8 Table. Result of multiple logistic regressions of moderate and severe pain across covariates (with the exposure to TE category: war-related)

|  | **Multivariable analysis** | | |
| --- | --- | --- | --- |
|  | **AOR (95% CI)** | | |
|  | **Total** | **Female** | **Male** |
| **War-related** | 1.06 (0.71-1.57) | 1.09 (0.64-1.80) | 1.06 (0.56-1.93) |
| **Age** |  |  |  |
| 40-49 | Ref. | Ref. | Ref. |
| 50-59 | 1.85 (1.17-3.01) | 1.93 (1.11-3.47) | 1.66 (0.73-4.12) |
| 60-69 | 2.25 (1.38-3.75) | 2.71 (1.51-5.01) | 1.47 (0.59-3.82) |
| 70-79 | 2.42 (1.48-4.02) | 2.23 (1.21-4.22) | 2.53 (1.13-6.23) |
| **Sex** |  |  |  |
| Female vs male | 1.74 (1.26-2.42) |  |  |
| **Country of origin** |  |  |  |
| Mozambique/other vs South Africa | 0.94 (0.64-1.34 | 0.91 (0.57-1.42) | 0.96 (0.49-1.77) |
| **Father’s occupation** |  |  |  |
| Skilled vs unskilled | 1.15 (0.80-1.68) | 1.01 (0.66-1.57) | 1.70 (0.83-3.84) |

*Note: Results are from multiple logistic regressions. The multivariable analysis was adjusted for age, sex, country of origin and father’s occupation. Sex was stratified in other two multivariable analyses. OR= odds ratio; AOR: adjusted odds ratio; CI= confidence interval*
